# Intrathecal tramadol added to bupivacaine as spinal anesthetic increases analgesic effect of the spinal blockade after major gynecological surgeries

**DOI:** 10.4103/0253-7613.43166

**Published:** 2008-08

**Authors:** Susmita Chakraborty, Jayanta Chakrabarti, Dipasri Bhattacharya

**Affiliations:** Department of Pharmacology, Institute of Postgraduate Medical Education and Research, Kolkata, India; 1 Department of Anesthesiology, Bangur Institute of Neurosciences and Psychiatry, Kolkata, India; 2 Bankura Sammilani Medical College and Hospital, Bankura, India

**Keywords:** Bupivacaine, gynecological surgeries, intrathecal administration, post operative analgesia, tramadol

## Abstract

The analgesic effect of the centrally acting opioid, tramadol, is well-known. It has been shown in clinical studies that using tramadol epidurally can provide longer duration of analgesia, without the common side effects of opioids. The study was undertaken to evaluate the duration of analgesia and/or pain free period produced by intrathecal tramadol added to bupivacaine in patients undergoing major gynecological surgery in a randomized double blind placebo controlled protocol. Fifty patients ASA I & II scheduled for Wardmayo's operation and Fothergill's operation were randomly allocated to two equal groups. Group A (n=25) received 3 ml of 0.5% hyperbaric bupivacaine (15 mg) with 0.2 ml of normal saline and Group B (n=25) received 3 ml 0.5% hyperbaric bupivacaine and 0.2 ml (20 mg) tramadol by intrathecal route at L3-4 inter space. Standard monitoring of the vital parameters was done during the study period. Levels of sensory block and sedation score were recorded every two minutes for the first 20 minutes, and then every ten minutes for the rest of the surgical procedure. Assessment of pain was done using Visual Analogue Scale (VAS). The study was concluded when the VAS was more than 40 mm, postoperatively. The patient was medicated and the time was recorded. Duration of analgesia or pain free period was estimated from the time of completion of spinal injection to administration of rescue analgesic or when the VAS score was greater than 40 mm. In Group B patients, the VAS score was significantly lower, as compared to Group A patients. The duration of analgesia was 210 ± 10.12 min in Group A; whereas, in Group B, it was 380 ± 11.82 min, which was found to be significant.

## Introduction

Spinal anesthesia with 0.5% hyperbaric bupivacaine is routinely administered nowadays for lower abdominal and major gynecological surgery. To increase the duration of analgesia produced by local anesthesia, a number of adjuvants have been added through the central neuraxial route. Intrathecal opioid administration has been demonstrated to provide effective postoperative analgesia after a variety of surgical procedures, at the cost of increased risk of respiratory depression.[1] Tramadol, in contrast to a centrally acting opioid analgesic, has minimal respiratory depressant effect,[2,3] because it has 6000 fold less affinity for *µ* receptors compared to morphine.[4,5] It also inhibits serotonin and norepinephrine reuptake in the spinal cord and has no reported neural toxicity.[6] Therefore, tramadol has the potential to provide effective postoperative analgesia, with no risk of respiratory depression after central neuraxial administration. However, pruritus, nausea, vomiting, urinary retention, activation of herpes labialis[7] and risk of unpredictable respiratory depression[8,9] have directed the clinicians to use a lower dose of tramadol that can be used intrathecally to produce effective and prolonged analgesia without such complications.

Therefore, this study was undertaken, in a randomized double blind placebo controlled protocol, to assess the effects of intrathecally administered tramadol with bupivacaine, on the duration of post operative analgesia in patients undergoing major gynecological surgeries.

## Materials and Methods

After approval by the Institutional Ethical Committee and written informed consent, 50 patients of physical status ASA (American Society of Anaesthesiologists) - I and II, aged between 45 and 60 years, scheduled for major gynecological surgery under spinal anesthesia, were enrolled in this study. Patients with a history of nonsensitivity to the drugs used, gross spinal deformity, peripheral neuropathy or having contraindication to regional anesthesia were excluded from this study.

All the patients received oral diazepam 10 mg at night, before the operation. Visual Analouge Scale (VAS), consisting of 100 mm line with 0 = no pain and 100 = worst possible pain, was explained to all the patients in their preoperative visit.

In the operating room, each patient received intravenous hydration with Ringer's lactate solution (20 ml/kg), before the induction of spinal anesthesia. Pulse rate, blood pressure, respiration rate, oxygen saturation and ECG monitoring were done in each patient and recorded before the induction of spinal anesthesia and thereafter during the procedure. A second anesthetist, who was otherwise uninvolved in the study, prepared the spinal injection solution. The anesthetist performing the block was blind to the solution administered and to the postoperative observations. Spinal anesthesia was carried out in right lateral position, with 25 G Quencke needle at L_3-4_ inter space, by a standard technique. The patients were randomly allocated to two groups - Group A (n=25) and Group B (n=25). Solutions were prepared with the help of disposable insulin syringe to measure 0.2 ml (20 mg) of preservative free tramadol and 0.2 ml normal saline. After free flow of cerebrospinal fluid from each patient after standard procedure of spinal anesthesia, Group A patients received 3 ml (15 mg) of 0.5%, hyperbaric bupivacaine and 0.2 ml normal saline. Group B patients received 3 ml (15 mg) of 0.5% hyperbaric bupivacaine and 0.2 ml (20 mg) of preservative free tramadol. The time of the intrathecal injection was noted and the patients were put in lithotomy position. Oxygen supplementation (4l/min) was administered with a facemask to all the patients during the operation. Following confirmation of spinal block by loss of sensation to cold and pinprick up to T_6-7_ level, surgery was allowed to start. The level of sensory anesthesia, defined as loss of sensation to pin prick test, was recorded bilaterally with a short bevelled needle at 2-min interval for the first 20 minutes and then at intervals of 10 minutes, until the end of surgery. Postoperatively, VAS score was noted every 30 minutes for six hours and the time was recorded when the VAS score was 40 mm or when the concerned patient demanded rescue analgesic in the form of intramuscular diclofenac sodium.

The duration of analgesia in Group A and B was obtained from the completion of spinal injection to the time of rescue analgesic administered on demand or when the VAS score was ≥ 40 mm. Sedation score (awake = 0, sleeping comfortably but easily arousable = 1, deep sleep but arousable = 2, deep sleep not arousable = 3) was noted every two minutes for 20 minutes, then every 10 minutes, till the end of surgery. Hypotension (defined as decrease in systolic blood pressure more than 20% of the base line value or less than 90 mm of Hg) after spinal injection was treated by increasing the rate of intravenous fluid administration and/or 5-10 mg of intravenous administration of bolus dose of ephedrine hydrochloride as and when required. Bradycardia (heart rate < 60 b.p.m) was treated with intravenous atropine 0.2 mg as and when needed. All the parametric data were analyzed by Student's t test and nonparametric data by Chi-square test, by a professional statistician and the result was considered to be significant (*P* < 0.05).

## Results

**Figure 1:**
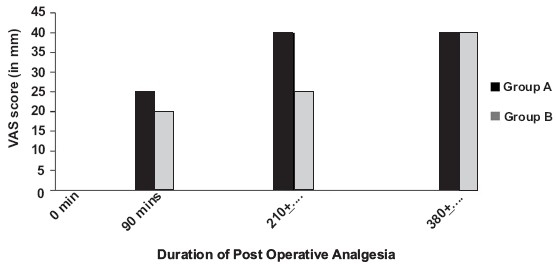
Comparison of VAS score between the two groups

The two groups - Group A and Group B - were comparable with respect to age, height, weight and duration of surgery [Table 1]. The duration of analgesia or pain free period in Group A was 210 ± 10.12 min, whereas, in Group B, it was 380 ± 11.82 min, as shown in Table 2. No clinically significant changes were observed in the heart rate, blood pressure, respiratory rate and sedation score in each of the two groups, intra operatively and/or postoperatively [Table 3]. A higher VAS score (≥ 4) was observed in Group A, whereas Group B patients showed significantly lower VAS score (< 4) more than six hours after the intrathecal injection. None of the patients had any postoperative complications like pruritus, vomiting, respiratory depression and lower limb weakness [Figure 1].

**Table 1:** Demographic profile of the two groups

	*Group A 0.5% hyperbaric bupivacaine with 0.2 ml normal saline*	*Group B 0.5% hyperbaric bupivacaine and 0.2 ml (20mg) tramadol*
Age (years)	46 ± 5.82	48 ± 2.23
Weight (in kg)	51 ± 2.3	49 ± 2.21
Height (in cm)	151 ± 3.2	152 ± 4.8
Duration of surgery (in min)	120 ± 10.23	118 ± 8.83

**Table 2:** Comparison of duration of analgesia between Group A and Group B patients

	*Group A n= 25 0.5 % hyperbaric bupivacaine with 0.2 ml normal saline Mean ± S.D.*	*Group B n= 25 0.5 % hyperbaric bupivacaine and 0.2 ml (20mg) tramadol Mean ± S.D.*
Duration of pain free period (in min)	210 ± 10.12	380 ± 11.82*

* *P* < 0.05

**Table 3:** Characteristics of hemodynamic and incidence of side effects among two groups during the study period

	*Group A n= 25 0.5% hyperbaric bupivacaine with 0.2 ml normal saline*	*Group B n= 25 0.5% hyperbaric bupivacaine and 0.2 ml (20mg) tramadol*
Bradycardia (heart rate < 60 b.p.m)	3 patients	4 patients
Hypotension (fall of b.p. > 20% from baseline)	8 (40%)	9 (45%)
Respiratory rate/min		
Mean ± S.D.	12 ± 2.3	13 ± 1.2
Sedation score		
Mean ± S.D.	2 ± 0.5	2 ± 0.2

## Discussion

Tramadol is a centrally acting analgesic agent with a terminal elimination half-life of 5.5 hours and provides clinical analgesia for 10 hours after epidural administration.[10,11] Tramadol stimulates the *µM*- receptors and to a lesser extent the δ and κ receptors. It also activates spinal inhibition of pain by decreasing the reuptake norepinephrine and serotonin. Although tramadol is one-fifth as potent as morphine as an analgesic, it causes less respiratory depression and pruritus. It was suggested by other studies that tramadol may have local anesthetic effects on peripheral nerves.[6] The effective dose range of intrathecal tramadol for postoperative analgesia is confusing until date. Therefore, our study was performed to demonstrate that intrathecal administration of 20 mg of tramadol when used with 0.5% bupivacaine prolonged the postoperative analgesia in the patients without producing related side effects like nausea, vomiting, pruritus and respiratory depression. A larger dose was avoided to prevent the increased incidence of postoperative sedation, nausea and vomiting, and a lower dose would not have the desired postoperative analgesic effects.

The result of the study showed that the duration of analgesia provided by intrathecal administration of 20 mg tramadol with 15 mg of 0.5% hyperbaric bupivacaine was significantly longer than that provided by intrathecal bupivacaine alone. The incidence of hemodynamic side effects like decreased blood pressure, bradycardia, and other side effects like somnolence and dryness of mouth were minimum and well tolerated by the patients studied. The respiratory rate of the patients also remained unaffected.

In conclusion, this study has demonstrated that tramadol (0.25 mg /kg body weight) when used with 0.5% hyperbaric bupivacaine intrathecally, significantly prolongs postoperative analgesia after major gynecological surgeries.
